# Pharmacologic Strategies for Intraoperative Hypotension When Ephedrine Is Unavailable: An Evidence-Based Review

**DOI:** 10.3390/jpm16070384

**Published:** 2026-07-17

**Authors:** Gilberto Duarte-Medrano, Natalia Nuño-Lámbarri, Diana Chavez-Muñoz, Rebeca Garazi Elguezabal Rodelo, Octavio Gonzalez-Chon, Luigi La Via

**Affiliations:** 1 Department of Anesthesia, Hospital Medica Sur, Mexico Puente de Piedra 150, Toriello Guerra, Tlalpan, Mexico City 14050, Mexico; 3c.chavezdiana@gmail.com (D.C.-M.); ogchon@medicasur.org.mx (O.G.-C.); 2 Translational Research Unit, Medica Sur Clinic & Foundation, Puente de Piedra 150, Toriello Guerra, Tlalpan, Mexico City 14050, Mexico; 3 Department of Surgery, Faculty of Medicine, The National Autonomous University of Mexico (UNAM), Escolar 411A, Copilco Universidad, Coyoacán, Mexico City 04510, Mexico; 4 Department of Anesthesia and Intensive Care 1, University Hospital Policlinico “G. Rodolico–San Marco”, 95123 Catania, Italy; luigi.lavia@unict.it

**Keywords:** intraoperative hypotension, vasopressors, ephedrine, norepinephrine, phenylephrine, perioperative hemodynamics, pharmacodynamics, anesthesia

## Abstract

**Background/Objectives**: Intraoperative hypotension (IOHs) affects up to 87% of patients under general anesthesia and is consistently associated with acute kidney injury, myocardial damage, stroke, and mortality. The intermittent unavailability of ephedrine across healthcare systems underscores the need for evidence-based alternatives. This review critically evaluates pharmacological options for IOH when ephedrine is unavailable, focusing on receptor pharmacodynamics, population-specific evidence, and clinical consequences of inadequately managed hypotension. **Methods**: A narrative, evidence-based review was conducted examining mechanisms of action, dosing strategies, adverse effect profiles, and clinical applicability of key vasoactive agents: ephedrine, phenylephrine, norepinephrine, and epinephrine. Population-specific evidence across obstetric, pediatric, and elderly cohorts was synthesized from randomized controlled trials, meta-analyses, and observational studies. The clinical impact of IOH on neurological, cardiovascular, and renal outcomes was reviewed. **Results**: Each vasopressor exhibits a distinct receptor-selectivity profile that determines its hemodynamic effect and optimal clinical context. Norepinephrine’s favorable α1/β1 balance tends to preserve cardiac output better than pure α1-agonists and has emerged as a promising alternative in obstetric and elderly populations, although the optimal agent ultimately depends on the underlying mechanism of hypotension and individual patient characteristics. Epinephrine provides combined vasopressor and inotropic support for hypotension with myocardial depression. IOH is associated with a greater than twofold increase in postoperative AKI and significantly elevated risks of myocardial infarction and stroke, with outcomes driven by cumulative hypotensive exposure rather than isolated pressure nadirs. **Conclusions:** Effective management of IOH requires individualized vasopressor selection guided by underlying pathophysiology, cardiovascular profile, and surgical context. A physiology-based strategy—rather than protocol-driven drug substitution—enables anesthesiologists to achieve precise hemodynamic control and preserve end-organ perfusion even when ephedrine is unavailable.

## 1. Introduction

In the current global landscape, management of perioperative hypotension faces not only clinical but also substantial logistical and pharmacological challenges. The irregular availability of essential vasoactive agents—such as ephedrine, norepinephrine, phenylephrine, and vasopressin—across different healthcare systems has compelled anesthesiologists to adopt alternative strategies. Those include peripheral administration of norepinephrine and the use of second-line agents with distinct pharmacodynamic profiles. In this setting, pharmacological competence and the ability to tailor hemodynamic management to resource limitations have become crucial skills for the contemporary anesthesiologist, ensuring both patient safety and therapeutic efficacy regardless of supply variability.

Perioperative hypotension is one of the most common and clinically significant hemodynamic disturbances, directly impacting tissue perfusion and postoperative outcomes. Its reported incidence may reach up to 87% of patients undergoing surgery under general anesthesia, and it has been consistently associated with an increased risk of acute kidney injury, myocardial damage, stroke, postoperative delirium, and early mortality [1,2]. From a pathophysiological standpoint, hypotension represents a disruption in the balance between cardiac output, systemic vascular resistance, and effective circulating volume. This interplay is influenced by anesthetic depth, surgical stress response, and patient comorbidities. Importantly, even brief reductions in mean arterial pressure (MAP) below 65 mmHg have been linked to end-organ dysfunction, particularly in patients with limited autoregulatory reserve or marginal baseline perfusion [3,4].

Despite an expanding body of literature, the operational definition of intraoperative hypotension (IOH) remains remarkably inconsistent. More than a hundred definitions have been reported, encompassing absolute thresholds (e.g., MAP < 65 mmHg or systolic pressure < 90 mmHg), relative thresholds (>20–30% reduction from baseline), or combined criteria incorporating both magnitude and duration of pressure decrease [5,6]. Although the Perioperative Quality Initiative (POQI-3) has proposed a practical definition (MAP < 65 mmHg sustained for more than one minute), the extent of organ injury appears to depend not solely on the pressure nadir, but also on the cumulative exposure below this threshold. Such methodological heterogeneity hampers cross-study comparability and the establishment of standardized therapeutic targets, underscoring the need for a physiologically grounded conceptualization of perioperative hypotension as a multifactorial and dynamic entity [7].

Within this framework, the present review aims to provide an integrative and critical evaluation of the pharmacological options currently available for the treatment of perioperative hypotension. Emphasis is placed on available pharmacological options for vasoactive agents, including their mechanisms of action, clinical limitations, and contextual applicability in varied healthcare environments, as well as evidence-based strategies for vasopressor selection. The clinical consequences of intraoperative hypotension are also reviewed. By addressing both evidence-based and pragmatic dimensions of hemodynamic management, this review seeks to support anesthesiologists in achieving individualized, physiology-guided, and resource-sensitive correction of hypotensive states in the perioperative setting.

## 2. Materials and Methods

This study constitutes a narrative, evidence-based review of the pharmacological management of intraoperative hypotension, with particular emphasis on therapeutic alternatives when ephedrine is unavailable. Given its narrative design, no formal registration, systematic protocol, or PRISMA-compliant methodology was applied.

A comprehensive, non-systematic literature search was conducted across PubMed/MEDLINE, Embase, and the Cochrane Library, covering publications from January 2000 through March 2026, with no language restrictions applied. The search strategy incorporated the following MeSH terms and free-text keywords in various combinations: intraoperative hypotension, perioperative hemodynamics, vasopressors, ephedrine, phenylephrine, norepinephrine, epinephrine, vasopressin, angiotensin II, adrenergic receptors, cardiac output, mean arterial pressure, obstetric anesthesia, pediatric anesthesia, elderly surgical patients, acute kidney injury, postoperative outcomes, and hemodynamic management.

Article selection was performed by the authors based on clinical relevance, methodological quality, and contribution to the review objectives. Priority was given to randomized controlled trials, prospective cohort studies, systematic reviews, and meta-analyses. Seminal pharmacological studies, authoritative narrative reviews, and contemporary clinical guidelines were also incorporated when deemed relevant to provide mechanistic context or practical guidance. No formal risk-of-bias assessment or quality scoring instrument was applied, consistent with the narrative design of this review.

Included studies addressed one or more of the following domains: (1) receptor-level pharmacodynamics of vasoactive agents used in intraoperative hypotension; (2) dosing strategies, routes of administration, and safety profiles of ephedrine, phenylephrine, norepinephrine, and epinephrine; (3) population-specific evidence in obstetric, pediatric, and elderly patients; and (4) perioperative and postoperative consequences of intraoperative hypotension, including neurological, cardiovascular, and renal outcomes. Studies focused exclusively on septic shock, cardiac surgery, or intensive care unit vasopressor management without relevance to the intraoperative context were excluded.

Title/abstract and full-text screening were performed independently by two reviewers (G.D.-M. and D.C.-M.); disagreements regarding eligibility were resolved by a third senior reviewer (N.N.-L.). No artificial intelligence tools were used for data extraction, synthesis, or interpretation.

This search yielded a total of 115 records. Titles and abstracts were screened, and studies were excluded if they were duplicates, involved animal models, consisted of editorials or letters without original data, or did not directly address pharmacological alternatives to ephedrine for the management of intraoperative hypotension in adult patients. Following this screening process, 64 articles met the eligibility criteria and were included in the narrative synthesis of the present review.

## 3. Results

### 3.1. Receptor-Level Pharmacodynamics of Vasopressors and Inotropes in Intraoperative Hypotension

A mechanistic understanding of receptor pharmacology is essential to guide rational vasopressor selection in intraoperative hypotension, particularly when ephedrine is unavailable. Contemporary evidence consistently emphasizes that the hemodynamic profile of vasoactive agents reflects a complex interplay between adrenergic (α1, β1, β2) and non-adrenergic pathways, including vasopressinergic (V1a/V2), renin–angiotensin (AT1R), and nitric oxide–cyclic guanosine monophosphate (NO–cGMP) signaling axes. Rather than a simplistic classification into “vasopressors” or “inotropes,” these agents should be conceptualized according to their receptor-specific effects on vascular tone, venous capacitance, cardiac performance, and regional perfusion [8,9]. Activation of α1-adrenergic receptors remains the principal mechanism underlying arterial and venous vasoconstriction. Through phospholipase C activation and intracellular calcium mobilization, α1 stimulation increases systemic vascular resistance while simultaneously reducing venous capacitance, thereby augmenting stressed volume and venous return. This dual effect is particularly relevant in anesthesia-induced vasoplegia, where relative hypovolemia and reduced vascular tone coexist. However, excessive α1-mediated vasoconstriction may compromise stroke volume and organ perfusion, especially in preload-dependent states [10].

In contrast, β1-adrenergic receptor activation enhances myocardial contractility, heart rate, and lusitropy via cyclic adenosine monophosphate (cAMP)-mediated pathways. While this supports cardiac output, it occurs at the cost of increased myocardial oxygen consumption and a higher propensity for tachyarrhythmias. β2-receptor stimulation induces vasodilation and may counterbalance α-mediated vasoconstriction, contributing to the heterogeneous hemodynamic profiles of mixed agonists.

Among commonly used agents, norepinephrine provides a favorable balance of predominant α1 and moderate β1 activity, allowing restoration of arterial pressure while preserving cardiac output. In contrast, phenylephrine and metaraminol, as near-pure α1 agonists, increase systemic vascular resistance effectively but may reduce stroke volume and cardiac output, limiting their utility in patients with impaired ventricular function or preload dependence. This distinction is critical when substituting for ephedrine, whose indirect and mixed adrenergic effects confer both vasoconstrictive and inotropic support [11].

Epinephrine, with dose-dependent β1, β2, and α1 effects, offers potent inotropic and vasopressor support but is associated with increased lactate production, tachycardia, and myocardial oxygen demand, making it less suitable for routine management of non-refractory intraoperative hypotension. Similarly, dopamine exhibits dose-dependent dopaminergic and adrenergic activity but is associated with unpredictable pharmacodynamics and a higher incidence of arrhythmias, limiting its contemporary role [12].

**Figure 1:**
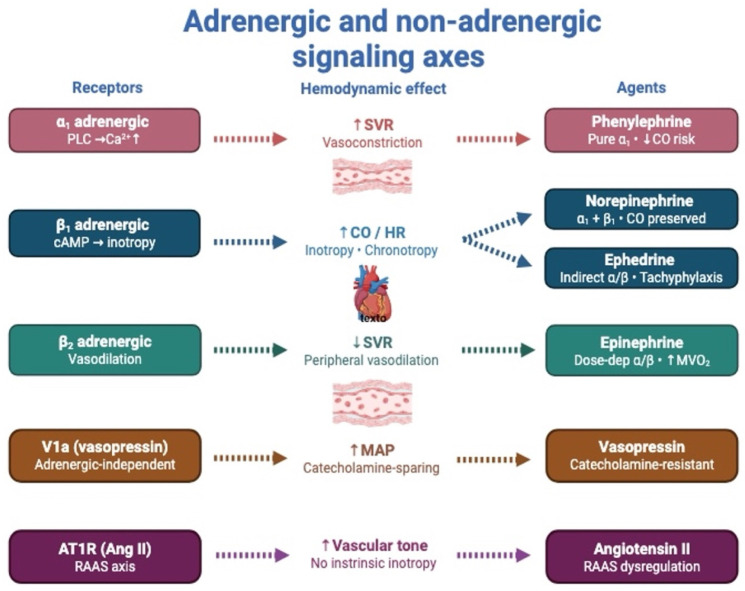
Receptor-Specific Hemodynamic Effects and Mechanistic Targets of Adrenergic and Non-Adrenergic Vasopressors.

Non-adrenergic pathways provide important adjunctive or rescue strategies. Vasopressin, acting primarily via V1a receptors, induces vasoconstriction independent of adrenergic receptors and is particularly effective in catecholamine-resistant vasoplegia. Its minimal direct cardiac effects may preserve myocardial oxygen balance; however, excessive vasoconstriction may impair splanchnic and coronary perfusion. Angiotensin II, via AT1 receptor activation, represents another non-catecholaminergic mechanism to restore vascular tone, particularly in states of renin–angiotensin system dysregulation, although it lacks intrinsic inotropic properties [13], see Figure 1.

### 3.2. Pharmacological Options

This section reviews the main pharmacologic strategies available for its management, focusing on the nature and mechanisms of action of commonly used agents, their receptor selectivity, dosing regimens, routes of administration, and safety considerations. Attention will be given to the comparative pharmacodynamics of adrenergic and non-adrenergic vasopressors, their impact on cardiac output and systemic vascular resistance, and the emerging evidence supporting context-specific approaches such as individualized blood pressure targets and peripheral vasopressor administration.

#### 3.2.1. Ephedrine

Ephedrine is a mixed-acting sympathomimetic amine that increases arterial pressure through both direct adrenergic receptor stimulation and indirect enhancement of endogenous catecholamine activity. It stimulates α_1_-, β_1_-, and β_2_-adrenergic receptors while simultaneously promoting presynaptic norepinephrine release and inhibiting its reuptake [14]. The resulting hemodynamic effect reflects a combination of increased systemic vascular resistance and augmented cardiac output. In contrast to pure α-agonists, the rise in MAP is largely mediated by enhanced heart rate and myocardial contractility rather than isolated vasoconstriction, making ephedrine particularly effective in hypotension accompanied by bradycardia or reduced cardiac output [14,15,16].

This balanced adrenergic profile distinguishes ephedrine from direct α-adrenergic agents such as phenylephrine, which commonly produce reflex bradycardia and a reduction in cardiac output. By preserving or increasing heart rate, ephedrine maintains forward flow while restoring perfusion pressure. However, its β_1_-adrenergic activity also increases myocardial oxygen consumption and may precipitate tachycardia or arrhythmias, limiting its suitability in patients with ischemic heart disease or significant ventricular dysfunction. A further limitation is tachyphylaxis, which develops with repeated administration. As presynaptic norepinephrine stores become depleted, the indirect sympathomimetic effect progressively diminishes. Consequently, although ephedrine is highly effective for transient intraoperative hypotension, its hemodynamic response becomes less predictable with sustained use [17].

In the intraoperative setting, ephedrine is typically administered as an intravenous bolus of 5–10 mg titrated to effect. The onset of action is rapid, and the duration generally ranges from 5 to 10 min. Continuous infusion is rarely employed due to the development of tachyphylaxis, and intramuscular or subcutaneous administration is uncommon in modern anesthesia practice [14,18]. Given these characteristics, ephedrine remains a useful agent for short-lived hypotensive episodes, particularly when preservation of cardiac output is desirable, whereas direct-acting vasopressors may provide more consistent control during prolonged hemodynamic instability.

#### 3.2.2. Phenylephrine

Phenylephrine is a selective α_1_-adrenergic receptor agonist that exerts its primary hemodynamic effect through arterial and venous vasoconstriction, leading to an increase in systemic vascular resistance and a consequent rise in MAP. Owing to the absence of β-adrenergic activity, phenylephrine possesses minimal intrinsic chronotropic or inotropic properties. Consequently, baroreceptor-mediated reflex bradycardia frequently follows abrupt elevations in arterial pressure, and cardiac output may decline in response to an increased afterload [19].

In the intraoperative management of hypotension, phenylephrine is typically administered as intravenous boluses of 50–100 µg (up to 250 µg), titrated according to clinical response. For sustained blood pressure support, continuous infusions of 0.5–1.4 µg/kg/min—prepared from a 10 mg/mL concentrate—are commonly employed and adjusted to individualized hemodynamic targets. Several formulations and institutional protocols describe fixed-rate adult infusions (e.g., 10–35 µg/min), with upper limits approaching 200 µg/min, highlighting the heterogeneity in practice and product labeling [20,21]. From a practical standpoint, phenylephrine offers distinct advantages. Its short duration of action and precise titratability allow for fine adjustment of arterial pressure via small, repeatable boluses, minimizing prolonged exposure to catecholamine infusions. Its exclusive α_1_-receptor activity renders it particularly valuable in scenarios where vasoconstriction is desirable without concomitant tachycardia—such as in neuraxial anesthesia-related hypotension or selected obstetric settings [22,23,24,25]. However, these same pharmacodynamic features also define its limitations. Reflex bradycardia and reductions in stroke volume and cardiac output are more pronounced with phenylephrine than with mixed or β-agonist agents such as ephedrine. Comparative physiologic studies consistently demonstrate lower heart rate and cardiac output during phenylephrine administration, despite restoration of MAP. Accordingly, phenylephrine may be suboptimal in patients with limited preload reserve, pre-existing bradyarrhythmias, or impaired ventricular function [20,26].

#### 3.2.3. Norepinephrine

Norepinephrine is an endogenous catecholamine characterized by potent α_1_-adrenergic agonism and moderate β_1_-adrenergic activity, with negligible β_2_ effect. Through α_1_-mediated constriction of arterial resistance and venous capacitance vessels, it increases systemic vascular resistance and MAP, while its β_1_ effects provide modest positive inotropy that helps support cardiac output. Although abrupt increases in MAP may provoke baroreflex-mediated vagal activation and relative bradycardia, heart rate is often maintained by concurrent β_1_ stimulation. In susceptible individuals, particularly those with impaired ventricular function or limited preload reserve, the associated increase in afterload may attenuate stroke volume [27,28,29].

In both intraoperative and critical-care settings, norepinephrine is administered as a continuous intravenous infusion, commonly initiated at 8–12 μg/min and titrated to a predefined MAP target. Maintenance rates frequently range from 2–4 μg/min, with higher doses reserved for refractory vasodilatory shock [28,30,31]. Weight-based dosing schemes (approximately 0.05–0.4 μg/kg/min) are also widely employed and adjusted in small increments according to hemodynamic response [32]. Central venous administration is preferred to reduce the risk of extravasation and local ischemic injury, although peripheral initiation may be acceptable when continuous site monitoring is ensured [33].

Clinically, norepinephrine offers reliable restoration of vascular tone with relative preservation of cardiac output compared with pure α-agonists such as phenylephrine. Its rapid onset and short context-sensitive half-life permit precise titration and stable achievement of target MAP. However, excessive vasoconstriction may compromise regional perfusion, particularly in hypovolemic patients or those with microcirculatory dysfunction [34]. Additional concerns include arrhythmias and tissue injury from extravasation, reinforcing the importance of adequate volume assessment, vigilant monitoring, and appropriate vascular access selection.

#### 3.2.4. Epinephrine

Epinephrine is an endogenous catecholamine exhibiting dose-dependent α- and β-adrenergic receptor agonism. At lower infusion rates, β_1_ and β_2_ effects predominate, resulting in increased myocardial contractility and heart rate together with peripheral vasodilation and bronchodilation. As doses escalate, α_1_-mediated vasoconstriction becomes increasingly dominant, producing elevation of systemic vascular resistance and MAP [9]. The overall hemodynamic response is therefore dynamic and context-dependent. While β_1_ stimulation frequently induces tachycardia and increases myocardial oxygen consumption, abrupt rises in MAP may also trigger baroreceptor-mediated vagal reflexes, occasionally resulting in transient bradycardia [35].

In perioperative and shock states, epinephrine is typically administered as a titrated intravenous infusion, most commonly at 0.02–0.1 μg·kg^−1^·min^−1^. Broader dosing ranges (0.05–2 μg·kg^−1^·min^−1^) are described in severe shock syndromes, with adjustments guided by predefined hemodynamic targets. Owing to its short plasma half-life, steady-state conditions are achieved rapidly. For transient circulatory support, push-dose epinephrine (5–20 μg IV boluses every 1–5 min using a 10 μg/mL dilution) may serve as a temporizing measure while definitive interventions are implemented [36,37,38,39]. Central venous infusion is preferred when feasible, although carefully monitored peripheral administration is acceptable.

The main advantage of epinephrine lies in its combined vasopressor and inotropic effects, enabling simultaneous restoration of vascular tone and augmentation of cardiac output. This profile may be particularly advantageous in hypotension accompanied by myocardial depression or in distributive states where both contractility and vascular tone are impaired. Nevertheless, potent adrenergic stimulation also accounts for its limitations. Tachyarrhythmias, excessive chronotropy, and increased myocardial oxygen demand are common concerns, and pronounced α-mediated afterload elevation may reduce stroke volume in vulnerable patients [40]. The risk of tissue ischemia following extravasation further necessitates careful vascular access management and continuous hemodynamic surveillance.

#### 3.2.5. Theodrenaline/Cafedrine (Akrinor^®^)

In several European countries—most notably Germany, Austria, and Switzerland—a fixed 20:1 combination of cafedrine and theodrenaline (Akrinor^®^) has historically been among the most frequently administered vasoactive agents for anesthesia-related and prehospital hypotension, in continuous clinical use since 1963 [41]. Cafedrine, a theophylline-linked norephedrine derivative, provides a predominantly indirect sympathomimetic and inotropic effect, while theodrenaline, a theophylline-linked noradrenaline derivative, contributes a direct vasoconstrictive component; the combination raises MAP chiefly through increased cardiac preload, stroke volume, and cardiac output, with comparatively little change in systemic vascular resistance or heart rate [41]. This profile—pressure restoration achieved largely through augmented cardiac output rather than pure vasoconstriction—has made cafedrine/theodrenaline particularly popular for spinal-anesthesia-induced hypotension during cesarean delivery, where national surveys have identified it as the most commonly used vasoactive substance in German-speaking obstetric anesthesia practice [42].

More recently, however, the use of cafedrine/theodrenaline appears to be gradually declining in favor of norepinephrine, mirroring the broader international shift toward continuous, titratable norepinephrine infusions discussed throughout this review. A retrospective single-center cohort study comparing cafedrine/theodrenaline with phenylephrine for spinal-anesthesia-induced maternal hypotension highlighted limitations of the traditional reactive bolus approach with cafedrine/theodrenaline, including greater blood-pressure variability compared with prophylactic, titrated vasopressor strategies [43]. Because cafedrine/theodrenaline is not marketed outside a small number of European countries, clinical experience with it remains largely confined to the German-language literature and is therefore underrepresented in the predominantly norepinephrine- and phenylephrine-centered evidence base reviewed elsewhere in this article; nonetheless, its long track record illustrates that mixed direct/indirect sympathomimetic strategies achieving pressure restoration through combined preload and cardiac-output augmentation, rather than isolated vasoconstriction, may offer a valid physiological alternative to pure α1-agonism in regions where it remains available.

#### 3.2.6. Vasopressin

Beyond its established role as a rescue agent in catecholamine-refractory vasoplegia, accumulating evidence supports the earlier, adjunctive use of vasopressin alongside norepinephrine rather than reserving it exclusively for refractory cases. In vasodilatory shock, a systematic review and meta-analysis of nine randomized controlled trials found that adding vasopressin or its analogue terlipressin significantly reduced norepinephrine requirements compared with catecholamine therapy alone, without impairing cardiac output, and was associated with a modest reduction in mortality [44]. In the perioperative and cardiac surgical setting specifically, the VANCS randomized controlled trial found that first-line vasopressin, compared with norepinephrine, reduced the composite outcome of mortality or severe complications in patients with vasoplegic shock after cardiac surgery, with fewer episodes of atrial fibrillation and postoperative acute kidney injury [45]. A more recent systematic review of randomized trials comparing vasopressin-receptor agonists with norepinephrine specifically for perioperative hypotension similarly found that combining vasopressin with norepinephrine achieved higher mean arterial pressure than norepinephrine alone and was associated with shorter intensive-care-unit and hospital stays in cardiac surgical vasoplegia, supporting a norepinephrine-sparing, combination-based strategy over sequential rescue dosing [46]. Clinically, this argues for considering low-dose vasopressin (typically 0.01–0.04 U/min) as an early adjunct once modest norepinephrine doses are required, rather than waiting for high-dose catecholamine refractoriness, since earlier co-administration may limit cumulative catecholamine exposure and its associated arrhythmogenic and vasoconstrictive adverse effects.

Vasopressin also holds a distinctive and clinically important role in patients with pulmonary hypertension and right ventricular dysfunction, a population in which conventional α1-adrenergic vasopressors may be relatively disadvantageous. Unlike phenylephrine, which increases pulmonary vascular resistance, vasopressin acting via V1 receptors has been shown experimentally to increase systemic vascular resistance while causing relative pulmonary vasodilation, improving the transpulmonary pressure gradient and right ventricular performance in a rat model of pulmonary hypertensive crisis [47]. This differential systemic-versus-pulmonary vasoconstrictive profile has been exploited clinically: case reports describe the successful use of vasopressin to treat systemic hypotension and right ventricular failure following cesarean delivery in patients with idiopathic pulmonary arterial hypertension, with improved hemodynamic variables and no adverse effect on right ventricular function [48]. Contemporary obstetric and cardiac anesthesia reviews now recommend vasopressin, rather than pure α1-agonists, as a preferred vasopressor for treating systemic hypotension in parturients and surgical patients with pulmonary arterial hypertension, reserving norepinephrine as a reasonable alternative when additional inotropic support is required [49]. As with its use in vasoplegia, caution is warranted at higher doses (typically > 0.04 U/min), which may cause coronary and mesenteric vasoconstriction and, in some experimental models, impair right ventricular contractility directly; vasopressin should therefore be titrated to the lowest effective dose alongside invasive hemodynamic monitoring in this high-risk population.

Although vasopressors share the common goal of restoring arterial pressure, their pharmacodynamic profiles differ substantially in receptor selectivity, effects on cardiac output, and impact on regional perfusion. These distinctions are clinically meaningful, as the mechanism by which blood pressure is restored—whether through increased systemic vascular resistance, enhanced cardiac output, or both—directly influences organ perfusion and myocardial workload.

Accordingly, vasopressor selection should not be driven solely by MAP targets, but rather by the underlying pathophysiology and the patient’s individual characteristics. Factors such as ventricular function, preload status, susceptibility to arrhythmia, myocardial ischemia risk, and the etiology of hypotension must guide therapeutic choice. An individualized approach—grounded in physiologic principles and careful hemodynamic assessment—remains essential to optimize perfusion while minimizing iatrogenic harm in both perioperative and critical-care populations.

### 3.3. Population-Specific Evidence for Vasopressor Selection in Intraoperative Hypotension

Across diverse clinical populations (including obstetric, pediatric, and elderly patients), the prevention and management of anesthesia-related hypotension has progressively shifted toward physiology-guided vasopressor strategies. This represents a departure from uniform, reactive bolus therapy. Increasing evidence suggests that optimal vasopressor selection depends not only on restoring MAP, but also on preserving cardiac output, minimizing adverse effects, and aligning pharmacodynamics with population-specific physiology.

In obstetric anesthesia, high-quality randomized controlled trials and meta-analyses demonstrate that norepinephrine provides hemodynamic stability comparable to, and in several studies superior to, phenylephrine during spinal anesthesia for cesarean delivery. Norepinephrine is associated with improved preservation of maternal heart rate and cardiac output, reduced incidence of bradycardia, and comparable neonatal acid–base outcomes when compared with phenylephrine [24,50,51,52,53]. These findings have prompted reconsideration of phenylephrine as the default first-line agent in this population and support norepinephrine as a viable alternative in appropriately selected patients, particularly when preservation of maternal heart rate and cardiac output is a priority; the choice between agents should nonetheless remain guided by the predominant hemodynamic mechanism (e.g., vasoplegia versus reduced cardiac output) and individual maternal cardiovascular status rather than by a uniform preference for one agent.

In pediatric populations, particularly neonates and young infants, cardiovascular physiology differs substantially from adult physiology, with greater dependence on heart rate for cardiac output and limited capacity to augment stroke volume. Available data suggests that traditional adult-derived ephedrine dosing may produce inconsistent or attenuated responses in younger patients. Emerging observational and institutional practice reports indicate increasing use of norepinephrine infusions for anesthesia-related or vasodilatory hypotension, although standardized dosing regimens and high-quality randomized pediatric trials remain limited [54,55]. These findings underscore the need for age-adapted vasopressor strategies rather than extrapolation from adult paradigms.

**Figure 2:**
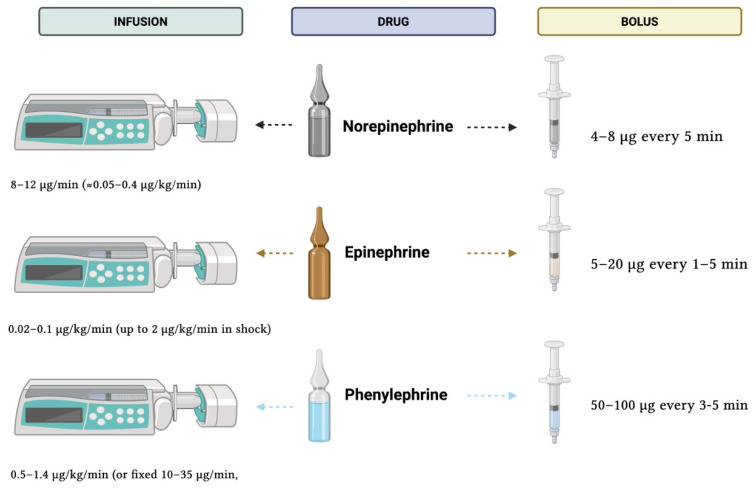
Recommended Bolus and Continuous Infusion Dosing Strategies for Common Vasopressors in the Management of Intraoperative Hypotension.

Among elderly patients, intraoperative hypotension is highly prevalent due to age-related reductions in autonomic responsiveness, impaired baroreflex sensitivity, and increased susceptibility to anesthetic-induced vasodilation. Prospective trials and cohort studies demonstrate that prophylactic or early low-dose norepinephrine infusions reduce the incidence and duration of hypotensive episodes, decrease total fluid administration, and may limit intraoperative blood loss without increasing adverse cardiac or renal outcomes [56,57,58,59]. Alpha-dominant vasopressors administered as controlled infusions appear to provide more stable hemodynamic profiles than intermittent bolus strategies in this population see Figure 2.

Collectively, these population-specific data support a paradigm shift away from traditional ephedrine-centered, reactive treatment of hypotension toward preventive, titrated, and physiology-based vasopressor administration. Rather than viewing vasopressors as interchangeable agents for MAP correction, current evidence favors individualized selection aligned with patient age, cardiovascular reserve, and procedural context. Study designs, patient characteristics, dosing strategies, and reported outcomes are summarized in Table 1.

### 3.4. Perioperative and Postoperative Consequences of Intraoperative Hypotension

A growing body of evidence has associated IOH with a spectrum of postoperative complications involving cerebral, cardiac, and renal systems, and, in severe or prolonged cases, increased mortality. However, defining a causal link and identifying universally applicable thresholds remain contentious, underscoring the complex interplay between individual physiology, surgical stress, and anesthetic management.

#### 3.4.1. Neurological and Cognitive Sequelae

Cerebral blood flow is tightly regulated through autoregulatory mechanisms that maintain perfusion across a range of MAP. When MAP falls below the lower limit of cerebral autoregulation, hypoperfusion and ischemia may occur, predisposing to neurological injury. Yu et al. [60] emphasize that IOH, although inconsistently defined across studies, can contribute to perioperative neurological injury, including stroke, postoperative delirium (POD), and postoperative cognitive dysfunction (POCD). While absolute thresholds such as MAP < 65 mmHg or relative decreases exceeding 20–30% from baseline have been frequently cited, the evidence indicates that the relationship is not uniform. Individual variability in cerebral autoregulation, surgical type, anesthetic agents, and patient comorbidities all modify the risk profile.

Yu and colleagues advocate for blood pressure management guided by cerebral oximetry (rScO_2_) or dynamic assessment of autoregulatory function, rather than relying on fixed thresholds, as such individualized strategies correlate with reduced postoperative neurocognitive complications.

#### 3.4.2. Cardiovascular and Renal Complications

From a cardiovascular perspective, hypotension compromises coronary perfusion pressure, potentially leading to myocardial ischemia, arrhythmias, or infarction, particularly in patients with limited coronary reserve. Rangasamy et al. [61]. conducted a detailed analysis in vascular surgical patients, revealing that the duration of IOH below MAP 65 mmHg was significantly associated with a higher incidence of composite postoperative complications—acute kidney injury (AKI), myocardial infarction, congestive heart failure, stroke, and death. Notably, relative decreases in MAP from baseline were not predictive of adverse outcomes, reinforcing the premise of duration and absolute perfusion thresholds over relative metrics. The deleterious effects were especially evident after 60 min of sustained hypotension, highlighting the temporal dimension as a determinant of postoperative morbidity.

Renal vulnerability to hypotension has also been extensively documented [62]. Hypoperfusion can impair glomerular filtration, particularly in the context of pre-existing renal insufficiency or atherosclerotic vascular disease. In large meta-analyses, including that by Cai et al. [63] IOH was associated with a more than twofold increase in postoperative AKI (OR 2.69; 95% CI 2.15–3.37) and significantly elevated risks of myocardial infarction (OR 2.11; 95% CI 1.41–3.16) and stroke (OR 1.33; 95% CI 1.21–1.46) after non-cardiac surgery. These findings underscore that IOH represents a systemic insult affecting multiple organ systems rather than an isolated hemodynamic disturbance.

#### 3.4.3. Mortality and Global Postoperative Outcomes

The meta-analysis of randomized controlled trials by D’Amico et al. [64] provides a valuable counterbalance to the prevailing observational evidence. Across more than 9000 surgical patients, no significant difference in mortality was observed between permissive (MAP ≤ 60 mmHg) and targeted (MAP > 60 mmHg) intraoperative management strategies. Interestingly, permissive hypotension was associated with a lower incidence of atrial fibrillation and a shorter hospital stay, suggesting that in certain contexts, moderate hypotension may be physiologically tolerable or even beneficial, provided that tissue perfusion is maintained and hypotensive episodes are not prolonged.

These findings challenge the linear assumption that lower intraoperative blood pressure uniformly translates to harm. Instead, they highlight that postoperative outcomes likely depend on the interaction between hypotension severity, duration, patient comorbidities, and the capacity for autoregulatory adaptation.

## 4. Discussion

The management of IOH has progressively shifted from empiric, drug-centered approaches toward a more nuanced, physiology-driven strategy. The present review highlights that the intermittent unavailability of ephedrine—long regarded as a cornerstone vasopressor in anesthetic practice—should not be viewed merely as a logistical constraint, but rather as an impetus to refine hemodynamic reasoning and individualized pharmacologic decision-making.

Current evidence clearly indicates that IOH is not a uniform clinical entity, but rather the final expression of a dynamic imbalance between cardiac output, systemic vascular resistance, intravascular volume, anesthetic depth, and patient-specific cardiovascular reserve. Consequently, restoration of MAP should not be pursued as an isolated numerical target. Instead, the choice of vasoactive therapy must be aligned with the predominant pathophysiological mechanism underlying hypotension. In this regard, mixed α/β-adrenergic agents such as norepinephrine or epinephrine may be advantageous in patients with reduced cardiac output or impaired myocardial performance, whereas pure α_1_-agonists like phenylephrine may be appropriate in states of vasoplegia when tachycardia is undesirable.

Importantly, population-specific data reinforces the absence of a universally optimal vasopressor. Obstetric, pediatric, and elderly orthopedic patients exhibit distinct autonomic profiles, vascular responsiveness, and tolerance to changes in preload and afterload. The growing preference for low-dose, continuous norepinephrine infusions across several of these populations reflects a broader shift toward more stable and predictable MAP control, reduced fluid loading, and improved preservation of cardiac output compared with intermittent bolus strategies; this preference is most justified when the predominant mechanism is vasoplegic, and it does not obviate the need to select agents such as phenylephrine or epinephrine when the clinical picture instead points to preload dependence or myocardial depression.

Beyond drug selection, the review underscores that the clinical impact of IOH is strongly influenced by the duration and cumulative exposure to hypotension rather than by isolated pressure nadirs. This observation supports the integration of advanced hemodynamic monitoring and individualized blood pressure targets, particularly in patients with limited autoregulatory reserve. Thus, pharmacologic therapy should be considered one component of a broader hemodynamic management strategy aimed at optimizing tissue perfusion rather than merely correcting arterial pressure values.

The evidence underlying these conclusions is nonetheless heterogeneous and, in several respects, limited. The operational definition of IOH varies markedly across the cited studies, spanning absolute thresholds, relative reductions from baseline, and combined magnitude-duration criteria, which complicates direct comparison of reported effect sizes and may partly explain divergent findings across trials. Much of the population-specific evidence, particularly in pediatric and elderly cohorts, derives from observational studies, single-center pilot trials, or institutional practice surveys rather than adequately powered randomized comparisons; conclusions in these populations should therefore be regarded as hypothesis-generating rather than definitive. Most head-to-head vasopressor comparisons have been conducted in obstetric spinal anesthesia, and extrapolation of norepinephrine’s favorable profile to general surgical or non-obstetric elderly populations rests on a comparatively smaller and more heterogeneous evidence base. Dosing regimens for norepinephrine and epinephrine differ substantially between studies and institutions, limiting the comparability of adverse-effect and efficacy data. Finally, as a narrative rather than systematic review, no formal risk-of-bias assessment was applied, and publication bias favoring positive findings cannot be excluded. These limitations do not undermine the overall physiological rationale presented here but indicate that the strength of evidence supporting specific agent choices varies considerably across the populations and clinical scenarios discussed.

## 5. Conclusions

Intraoperative hypotension is a multifactorial, patient-specific hemodynamic disturbance for which no single vasopressor is universally optimal. When ephedrine is unavailable, phenylephrine, norepinephrine, and epinephrine can each be used safely and effectively, provided the choice among them is matched to the predominant mechanism of hypotension, baseline cardiovascular function, and surgical context, rather than applied as interchangeable substitutes.

This physiology-based approach—as opposed to a fixed drug-substitution protocol—enables anesthesiologists to achieve more precise hemodynamic control, preserve end-organ perfusion, and potentially reduce postoperative complications. In an era of variable drug availability and increasing patient complexity, this capacity to tailor both the hemodynamic target and the pharmacologic intervention stands as a core competence of contemporary anesthetic practice and a cornerstone of safe, evidence-based perioperative care.

## Figures and Tables

**Table 1:** Pharmacological and clinical characteristics of each drug applied to the management of intraoperative hypotension.

Drug	Typical Dosing (Intraoperative/Acute)	Adverse Effects	Advantages	Disadvantages	Ideal Patient/Clinical Profile
Ephedrine	IV bolus: 5–10 mg, repeated as needed; IM/SC: 25–50 mg (longer duration); Infusion: rarely used due to tachyphylaxis.	Tachycardia, arrhythmias, ↑ <myocardial O_2_ demand, tachyphylaxis with repeated doses, possible ↑ afterload with ↓ stroke volume at high doses.	Increases MAP while maintaining/increasing HR and CO; precise titration with small boluses; widely available and inexpensive.	Efficacy diminishes with repeated administration; not reliable for prolonged hypotension or catecholamine-depleted patients.	Transient intraoperative hypotension with bradycardia or low cardiac output; short procedures requiring intermittent bolus therapy.
Phenylephrine	IV bolus: 50–100 µg (range 50–250 µg) titrated to response; Infusion: 0.5–1.4 µg/kg/min (or fixed 10–35 µg/min, up to 200 µg/min).	Reflex bradycardia, ↓ stroke volume, ↓ cardiac output, possible hypertension with excessive dosing.	Short-acting, precisely titratable, widely available; pure α_1_ agonist useful when tachycardia is undesirable.	Can markedly reduce HR and CO; less effective in preload-dependent or ventricular dysfunction states.	Neuraxial anesthesia-related hypotension, obstetric anesthesia, or cases requiring BP support without increasing HR.
Norepinephrine	IV bolus: 4–8 µg (range 4–16 µg) for transient hypotension; Infusion: start 8–12 µg/min (≈0.05–0.4 µg/kg/min) titrated to MAP goal; central line preferred, peripheral acceptable with monitoring.	Reflex bradycardia, arrhythmias, peripheral or splanchnic hypoperfusion, extravasation injury.	Highly controllable with rapid onset and offset; preserves HR/CO better than pure α-agonists; excellent MAP titration capability.	Afterload-mediated CO reduction in hypovolemia or poor ventricular function; requires careful monitoring and access.	Vasodilatory or neuraxial hypotension requiring continuous and precise pressure control; suitable when tachycardia should be avoided.
Epinephrine	IV bolus (push-dose): 5–20 µg every 1–5 min (10 µg/mL dilution) for transient hypotension; Infusion: 0.02–0.1 µg/kg/min (up to 2 µg/kg/min in shock) titrated to MAP or CO target.	Tachyarrhythmias, tachycardia, ↑ myocardial O_2_ demand, possible baroreflex bradycardia, afterload-induced ↓ stroke volume, local ischemia with extravasation.	Mixed α/β agonist supporting both vascular tone and contractility; rapid onset and short duration permit precise control; widely available.	Risk of arrhythmias and myocardial ischemia; excessive α activity may reduce CO at higher doses; requires central access for infusions.	Hypotension with myocardial depression or refractory vasodilatory hypotension

Note: ↑ = Increases; ↓ = Decreases.

## Data Availability

No new data were created or analyzed in this study. Data sharing is not applicable to this article.

## Abbreviations

The following abbreviations are used in this manuscript:

AKI	Acute Kidney Injury
Ang II	Angiotensin II
AT1R	Angiotensin II Type 1 Receptor
cAMP	Cyclic Adenosine Monophosphate
CO	Cardiac Output
HR	Heart Rate
IM	Intramuscular
IOH	Intraoperative Hypotension
IV	Intravenous
MAP	Mean Arterial Pressure
NO	Nitric Oxide
O_2_	Oxygen
OR	Odds Ratio
PICU	Pediatric Intensive Care Unit
POCD	Postoperative Cognitive Dysfunction
POD	Postoperative Delirium
POQI	Perioperative Quality Initiative
RAAS	Renin–Angiotensin–Aldosterone System
rScO_2_	Regional Cerebral Oxygen Saturation
SVR	Systemic Vascular Resistance
V1a	Vasopressin Receptor Type 1a
V2	Vasopressin Receptor Type 2
